# Combined transcriptome and metabolome analyses reveal the potential mechanism for the inhibition of *Penicillium digitatum* by X33 antimicrobial oligopeptide

**DOI:** 10.1186/s40643-021-00472-5

**Published:** 2021-12-02

**Authors:** Shuhua Lin, Yuanxiu Wang, Qunlin Lu, Bin Zhang, Xiaoyu Wu

**Affiliations:** 1 College of Bioscience and Bioengineering, Jiangxi Agriculture University, Nanchang, 330045 China; 2 Jiangxi Engineering Laboratory for the Development and Utilization of Agricultural Microbial Resources, Nanchang, 330045 China; 3 Collaborative Innovation Center of Postharvest Key Technology and Quality Safety of Fruits and Vegetables in Jiangxi Province, Nanchang, 330045 China

**Keywords:** X33 antimicrobial oligopeptide, *Penicillium digitatum*, Antimicrobial mechanism, Transcriptomics, Metabolomics

## Abstract

*Penicillium digitatum* is the primary spoilage fungus that causes green mold during postharvest in citrus. To reduce economic losses, developing more efficient and less toxic natural antimicrobial agents is urgently required. We previously found that the X33 antimicrobial oligopeptide (X33 AMOP), produced by *Streptomyces lavendulae* X33, exhibited a sterilization effect on *P. digitatum*. In this study, the effects, and physiological mechanisms of X33 AMOP as an inhibitor of *P. digitatum* were investigated. The transcriptional and metabolome profiling of *P. digitatum* exposed to X33 AMOP revealed 3648 genes and 190 metabolites that were prominently changed. The omics analyses suggested that X33 AMOP mainly inhibited *P. digitatum* growth by affecting cell integrity, genetic information delivery, oxidative stress tolerance, and energy metabolism. These findings provide helpful information regarding the antimicrobial mechanism of X33 AMOP against *P. digitatum* at the molecular level and indicate that X33 AMOP is a potential candidate to control *P. digitatum*.

**Graphical Abstract:**

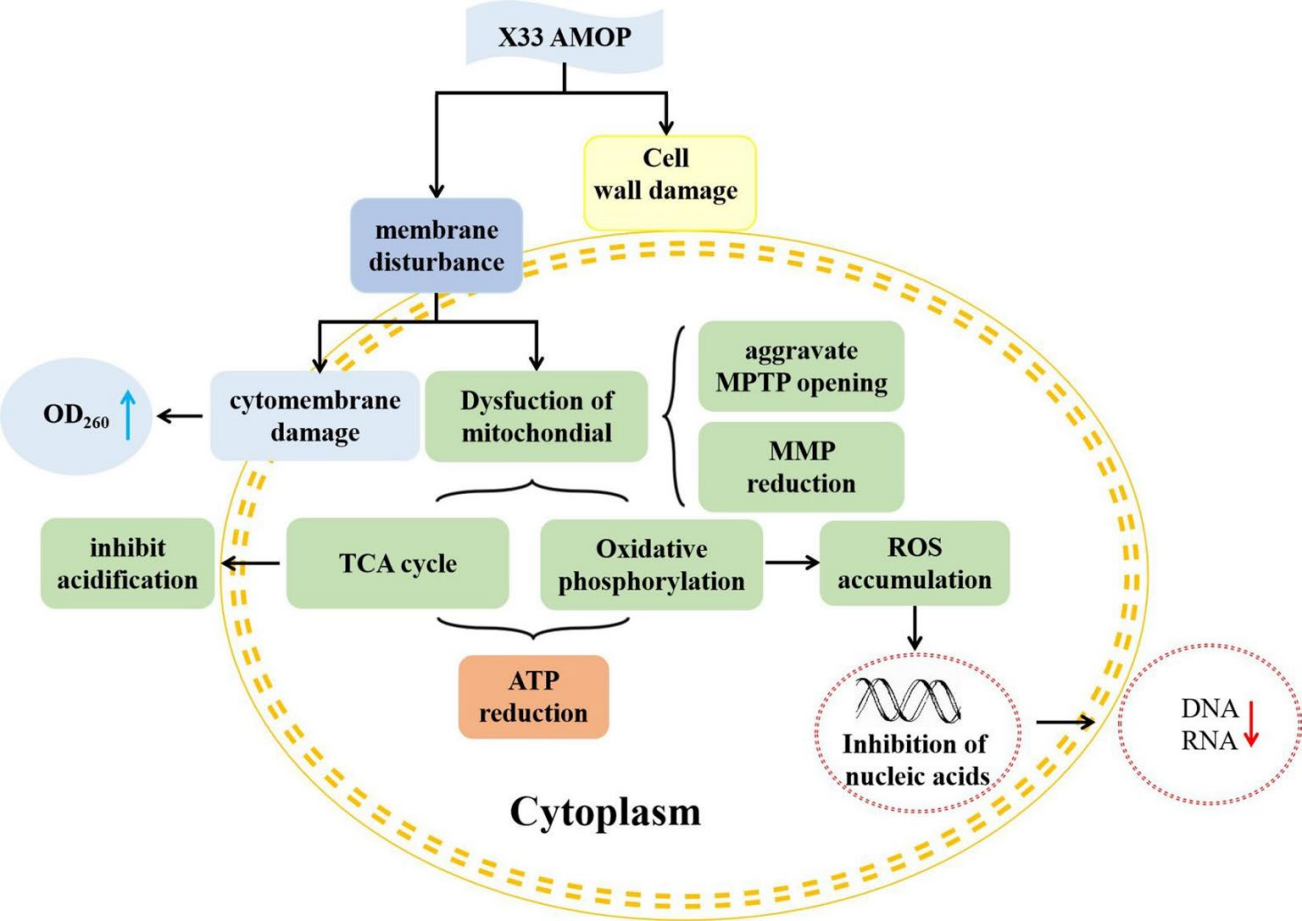

**Supplementary Information:**

The online version contains supplementary material available at 10.1186/s40643-021-00472-5.

## Introduction

The rapid increase in the occurrence of novel food-borne diseases caused by microbial spoilage is of great concern to humans because of the concerns regarding food safety. Simultaneously, microbial pollution has resulted in heavy economic damages in the food and agriculture industries. During the citrus harvest period, *Penicillium digitatum*, *P. italicum*, *Geotrichum citri-aurantii*, and *Aspergillus niger* are frequently found on the surface of the pericarp, causing significant economic losses by triggering green mold growth (Wang et al. 2020). To address this issue, chemical preservatives have been extensively used in the food industry. However, excessive application of chemical preservatives and synthetic fungicides causes major negative effects on human health due to their carcinogenic and teratogenic characteristics (Martínez-Blay et al. 2020).

Efforts to control fruit and vegetable diseases have been attempted for decades by developing alternative techniques, such as creating biological control agents and plant extracts, and synthesizing antimicrobial peptides (AMPs). AMPs are small peptides that are broadly found in microbes, insects, animals, and plants, and they have a wide spectrum of activity against parasites, fungi, bacteria, and viruses (Liu et al. 2019). Liu et al. (2019) showed that ε-poly-l-lysine efficiently controlled the disease progression of *Alternaria alternata* on tobacco plants by inhibiting spore germination and germ tube elongation, and downregulating the key genes involved in fungal development, and the peptide thanatin inhibited DNA, RNA, and protein biosynthesis, blocking *P. digitatum* growth. Due to their advantages of antifungal activity, lack of contamination, high level of drug resistance ability, extensive studies have been performed to improve the productivity and types of AMPs (Wang et al. 2018a). Microorganisms are a rich source of AMPs, consisting of numerous species and quantities. AMPs from microorganisms are a crucial source for the exploitation of novel antimicrobial agents for agricultural applications. In a previous study, we found that X33 antimicrobial oligopeptide (AMOP), a water-soluble ε-polylysine-analogous antimicrobial oligopeptide isolated from the fermentation liquor of the *Streptomyces lavendulae* strain X33 (CCTCC M2013163), had a strong bacteriostatic effect on *P. digitatum* (Lin et al. 2020). However, information regarding its mode of action on *P. digitatum* at the molecular level is limited and further investigation is required.

Recently, omics technology has been employed to assess the mechanism of pathogen–host interaction or pathogenic drug tolerance, including the study of fungal response mechanisms to AMPs. Allobawi et al. (Allobawi et al. 2020) showed that the combination of polymyxin B and ivacaftor inhibited *Pseudomonas aeruginosa* by downregulating the citrate cycle and glycolysis via metabolomic profiling analysis. In addition, the transcriptional profile of *P. digitatum* exposed to *Bacillus* cyclic lipopeptides was assessed to study the response mechanism (Tunsagool et al. 2019). The use of omics technology could promote a better understanding of the molecular mechanisms of pathogen–host interactions. Therefore, the present study aimed to determine the influence of X33 AMOP on the entire gene expression and metabolic profile of *P. digitatum* to understand the molecular mechanism and related pivotal pathways.

## Materials and methods

### Strains and reagents

The culture of *S. lavendulae* strain X33 was undertaken in a 150-mL conical flask containing 30 mL of fermentation medium (20 g of soluble starch, 3 g of beef extract, 10 g of fish meal peptone, 0.6 g of NH_4_Cl, 10 g of NaCl, and 0.02 g of CaCO_3_; initial pH 7.0; and 1 L of ultrapure water, pH 7.0), inoculated with a 1 mL spore fluid (10^7^ CFU/mL), and cultivated at 28 °C on a rotary shaker at 180 rpm for 96 h (Lin et al. 2020). X33 AMOP was separated from the fermentation broth of *S. lavendulae* strain X33 (stored in a typical culture collection center in China, No. CCTCC M2013163) following the methodology described in our previous study. *P. digitatum* Pd165 was isolated from decayed Nanfeng tangerine in our laboratory.

### Transcriptome assay

Strain Pd165 spores were inoculated in potato dextrose broth cultured at 28 °C and 160 rpm for 3 days. Different concentrations (0, the ck group; and 1.2 g/L, the sy group) of the X33 AMOP were added to the fermentation cultures at 2 days. After vacuum filtration, the mycelia were rinsed with sterile water and collected. The collected hyphae were rapidly frozen using liquid nitrogen for total RNA preparation, RNA quality inspection, cDNA library construction, and RNA-seq, which was conducted at Major Biomedical Co., Ltd. (Shanghai, China). Total RNA was derived using Omega reagent (Bio-Tek, USA) according to the manufacturer’s instructions. The cDNA libraries were constructed based on previous studies and sequenced on the Illumina Novaseq6000 platform (Lai et al. 2017). The obtained RNA-seq reads were mapped onto the reference genome of *P. digitatum* PHI26 (GCA_000315665.1) using Bowtie 2 v2.4.1. Subsequent analyses, including the quantification of gene expression (kallisto v0.46.2), verification of differentially expressed genes (DEGs) (DESeq2 v1.24), enrichment assay of DEGs by GO-enriched analysis (GOAtools v0.6.5), and KEGG pathway assay (KOBAS v2.1.1), were conducted following the methodologies described in a previous study (Wang et al. 2016).

### Real-time fluorescence quantitative polymerase chain reaction (qRT-PCR)

A total of 10 DEGs were chosen to verify the transcriptome data. The RNA and corresponding cDNA templates used in the qRT-PCR analysis were prepared using Omega reagent (Bio-Tek) based on the manufacturer’s instructions (OuYang et al. 2016). All primer pairs used for the expression analyses are presented in the supporting material (Additional file 1: Table S1). The reaction procedure was programmed as follows: 95 °C for 10 min, followed by 40 cycles at 95 °C for 15 s, and 60 °C for 30 s. The 2−^△△CT^ approach was applied to quantify the value of each specimen using the actin (PDIP_27720) gene as an internal reference.

### Metabolomics assay

After X33 AMOP treatment, a metabolomics assay of *P. digitatum* was conducted as described previously (Guo et al. 2020). Each group was analyzed using six biological replicates. Briefly, the samples were homogenized in water and blended with methanol acetonitrile solution (1:1, v/v). Then, the samples were homogenized twice with cryogenic ultrasonication for 0.5 h. The samples were centrifuged at 4 °C for 20 min after being deposited for 60 min at −20 °C to precipitate the proteins. The supernatant was harvested and freeze-dried. Prior to the liquid chromatography–mass spectrometry (LC–MS) assay, the samples were dissolved in acetonitrile aqueous solution (acetonitrile/water = 1:1, v/v), and were then assayed on an ultra-high performance liquid chromatography (UPLC; Agilent 1290 Infinity LC) coupled with an electrospray ionization quadrupole time-of-flight mass analyzer (AB Sciex Triple TOF 6600). The UPLC contained a Waters ACQUITY UPLC HSS T3 C18 column (2.1 × 100 mm, 1.8 µm) at a 0.4 mL/min flow rate with an acetonitrile gradient in 25 mM ammonium acetate and 25 mM ammonium hydroxide (0%−95% in 12 min). The information-dependent acquisition was applied in positive and negative modes. To monitor the stability and repeatability of the instrumental assay, the quality control sample consisted of all samples mixed.

Data were obtained and analyzed using the XCMS software. Peak lists were generated from MS/MS spectra acquired between 10 and 60 min, with a filtering noise threshold at 2% maximal intensity. Metabolite annotation was attempted based on the precise mass of the molecules (< 25 ppm), followed by their secondary spectral pattern in the in-house database. The processed information was subjected to data assay after normalization to the total peak intensity. The variable importance in the projection (VIP) value of each metabolite in the orthogonal partial least squares-discriminant analysis model was calculated to reveal its contribution to classification. Metabolites with VIP > 1 and *P* < 0.05 were considered different metabolites.

### Detection of the release of cellular elements

The release of cell components was assayed following a previously described methodology with some modifications (Wang et al. 2018b). Briefly, *P. digitatum* hyphae from 50 mL of the PDB broth were harvested by centrifugation at 12,000×*g* and 4 °C for 15 min, rinsed with sterilized water, and resuspended in 0.85% normal saline. The suspensions were then treated with X33 AMOP at various concentrations of 0, 1.2, 2.4, and 4.8 g/L for 2 h, respectively. Subsequently, 200 µL of supernatant was used to assay the absorbance at 260 nm using a microplate reader (Varioskan LUX, Thermo Fisher Scientific, USA).

### External medium acidification assays

Acidification of the extracellular medium was conducted as described previously (Tian et al. 2012). Hyphae (1.0 g) were resuspended in 40 mL of 50 mM KCl and refrigerated at 4 °C overnight. X33 AMOP was added to the suspension to obtain final concentrations of 0, 1.2, 2.4, and 4.8 g/L, and the treatment was performed for 0.5 h. After filtration, the hyphae were treated with 20 mL of 10% dextrose solution. The pH value was measured every hour.

### Determination of mitochondrial membrane potential (MMP)

The MMP was monitored according to the methodology described previously with minor modifications (Hong and Liu 2004). MMP disruption is associated with a lack of rhodamine-123 retention and a decrease in fluorescence. The separated mitochondria from the hyphae in the 1.2 g/L X33 AMOP treatment (without X33 AMOP treatment as the control) were incubated with rhodamine-123 in the assay medium (225 mM sucrose, 8 mM Tris–HCL, 13 mM K_2_HPO_4_, 10 mM KH_2_PO_4_, 5 mM MgC1_2_, 20 mM KCl, 5 μM rotenone, and 5 mM sodium succinate; pH 7.4) and continuously stirred. The fluorescence intensity was monitored at room temperature using a multiscan spectrum (Varioskan LUX, Thermo Fisher Scientific), with an excitation wavelength of 488 nm and an emission wavelength of 525 nm.

### Investigation of mitochondrial permeability transition pore (MPTP) opening

The MPTP opening was evaluated according to the methodology described previously with slight modifications (Yang et al. 2020). The isolated mitochondria from the hyphae with 1.2, 2.4, and 4.8 g/L of X33 AMOP treatment (or without X33 AMOP treatment as the control) were incubated with 2 mL of mitochondrial buffer (230 mM mannitol, 70 mM sucrose, and 3 mM HEPES; pH 7.4) at room temperature for 2 min. The absorbance of isolated mitochondria at 540 nm was subsequently measured and the MPTP opening was calculated using the following formula:

$${\text{MPTP}}\;{\text{opening}}/\% = \left( {{A_{540{\text{nm}}\;\text{control}}} - {\text{ }}{A_{540{\text{nm}}\;\text{treatment}}}} \right)/{A_{540{\text{nm}}\;\text{control}}}$$

### Assay of DNA and RNA content

The nucleic acids were quantified using the 4,6-diamidino-2-phenylindole (DAPI) binding approach, following the methodology of a previous study with minor modifications (Feng et al. 2020). DAPI is a fluorochrome that can eradiate blue fluorescence by effectually penetrating the cells and binding to the minor groove of the double-stranded DNA and the AU base pairs of RNA. The fresh spores were cultured for 12 h at 28 °C and subsequently treated with X33 AMOP (0, 1.2, 2.4, and 4.8 g/L) at 28 °C for 120 min. The 50-µL reactant mixture with an equal volume of DAPI was added to the well of a fluorescence plate and kept in the dark for 15 min. The fluorescence of DAPI binding to the DNA and RNA in the hyphae was separately monitored using a multiplate reader (Varioskan LUX, Thermo Fisher Scientific) at excitation wavelengths of 364 nm and 400 nm.

### Statistical analysis

Except for the omics assay results, all data are expressed as the mean ± standard deviation of three independent replicates. A one-way analysis of variance followed by Duncan’s test was performed to test the significance of differences between means obtained among the treatments at the 5% significance level using SPSS statistical software (SPSS Inc., Chicago, IL, USA).

## Results

### Transcriptome sequencing quality evaluation

Omics analysis is a powerful technique to identify DEGs and proteins and changes in metabolites in organisms. To explore the potential function mechanism of X33 AMOP against *P. digitatum*, a transcriptome assay was performed to evaluate the specific response at the mRNA level (Table 1). The RNA-seq results showed that, on average, 54.26 million and 52.50 million raw reads were separately acquired from the control (treated with sterilized water, the ck group) and treatment (treated with 1.2 g/L X33 AMOP, the sy group) groups, respectively. After filtering the adaptor sequences, we obtained an average of 53.81 and 52.06 million clean reads from the ck and sy groups, respectively. Among these, 92.85%–93.37% of the total clean reads from the ck and sy groups were aligned to the reference sequence (*P. digitatum* PHI26). Additionally, the mapped reads of all the six samples representing the filtered data were less than 1%. In summary, none of the sequencing samples were contaminated and all met the requirements for transcriptional analysis.

**Table 1:** Profile of the transcriptome sequence data

Parameter	ck	sy
Raw reads (million)	54.26	52.50
Clean reads (million)	53.81	52.06
Total mapped (%)	92.85	93.37
Error rate (%)	0.02	0.02
Q20 (%)	98.20	98.03
Q30 (%)	94.69	94.25
GC content (%)	52.96	52.58

### Functional classification and pathway analysis of DEGs

**Fig. 1:**
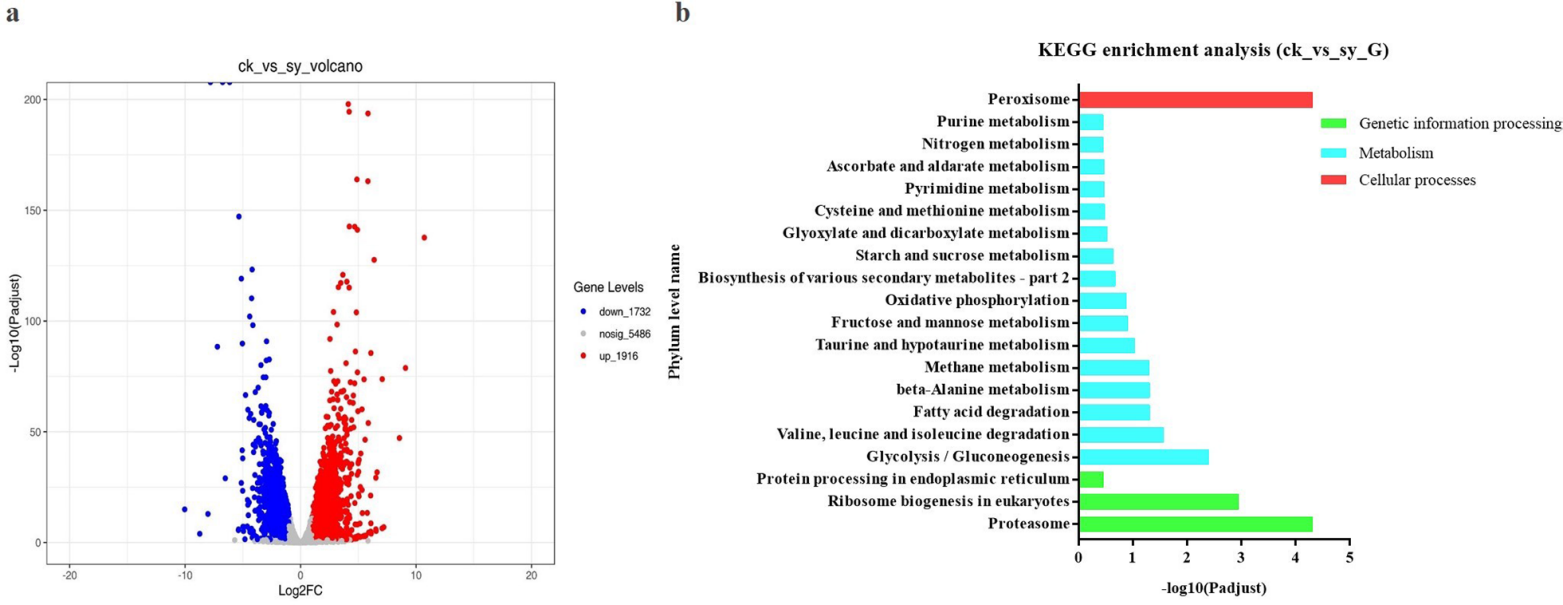
Differentially expressed genes (DEGs) enrichment analysis in *P. digitatum* cells under 1.2 g/L X33 AMOP treatment. **a** Cluster analysis volcano profile of DEGs. **b** Mostly enriched KEGG pathway of DEGs in *P. digitatum*. Ck represents the control group treated by water. Sy represents the tested group treated by 1.2 g/L X33 AMOP

The DEGs between the two libraries (ck and sy groups) offered clues to the molecular mechanisms involved in the *P. digitatum* response to X33 AMOP. In total, 3648 genes showed remarkable changes in abundance following X33 AMOP treatment (Table 2). The expression of 1732 DEGs was downregulated and 1916 DEGs was upregulated (Fig. 1a). Genes with different expression levels were correlated to a wide variety of regulatory and metabolic processes. In total, 1417 DEGs were mapped to the KEGG database and annotated to 119 pathways. The most abundant DEGs (48) were enriched in oxidative phosphorylation (map00190), 44 DEGs were enriched in ribosome biogenesis in eukaryotes (map03008), and 39 and 35 DEGs were enriched in protein processing in the endoplasmic reticulum (map04141) and glycolysis/gluconeogenesis (map00010), respectively (Fig. 1b). Therefore, these metabolic processes in *P. digitatum* were potential targets for X33 AMOP.

**Table 2:** Identification of differentially expressed genes in *P. digitatum* after X33 AMP treatment

Gene	Description	log2^(FC)^	Regulated type
XP_014531257.1	Exoglucanase type C (CBH1)	2.06	Up
XP_014538149.1	Chitin synthase A (CHS1)	2.49	Up
XP_014531529.1	C-14 sterol reductase ERG24	−2.23	Down
XP_014537276.1	C-8 sterol isomerase ERG2	−2.03	Down
XP_014537574.1	Sterol delta 5,6-desaturase ERG3	−2.01	Down
XP_014532172.1	14-Alpha sterol demethylase ERG11	−2.62	Down
XP_014537574.1	Sterol delta 5,6-desaturase ERG3	−2.01	Down
XP_014531227.1	C-3 sterol dehydrogenase ERG26	−2.18	Down
XP_014531760.1	Phosphoglycerate mutase	−3.59	Down
XP_014531016.1	Enolase	−2.58	Down
XP_014533781.1	Glyceraldehyde-3-phosphate dehydrogenase	−3.43	Down
XP_014534552.1	Malate dehydrogenase	−2.17	Down
XP_014537588.1	Malate dehydrogenase, NAD-dependent	−3.10	Down
XP_014535348.1	Cytochrome c oxidase polypeptide vib (COX6B)	−2.15	Down
YP_004221861.1	Cytochrome c oxidase subunit 1 (COX1)	2.86	Up
XP_002559972.1	Cytochrome c oxidase subunit 7A (COX7)	−2.29	Down
XP_014533465.1	Glutathione S-transferase (GST)	2.34	Up
XP_014539105.1	Transcription initiation factor iif	−2.05	Down
XP_014532620.1	Small subunit of nuclear cap-binding protein complex	−2.48	Down
XP_014535713.1	DNA polymerase delta subunit 4	−2.35	Down
XP_014534305.1	Basal transcription factors (CCNH)	−1.00	Down
XP_014534778.1	DNA repair protein Rad50	−1.29	Down
XP_014532480.1	Myo-inositol-phosphate synthase (INO1)	−1.05	Down
XP_014531425.1	Acyl carrier protein (NDUFAb1)	−1.39	Down
XP_014536276.1	Isocitrate dehydrogenase (IDH)	−1.47	Down
XP_014532714.1	Heat shock protein (HSP70)	1.62	Up
XP_014530804.1	Superoxide dismutase	−2.61	Down
XP_014530915.1	Catalase B	−1.98	Down

### Changes in *P. digitatum* metabolite response to X33 AMOP incubation

**Fig. 2:**
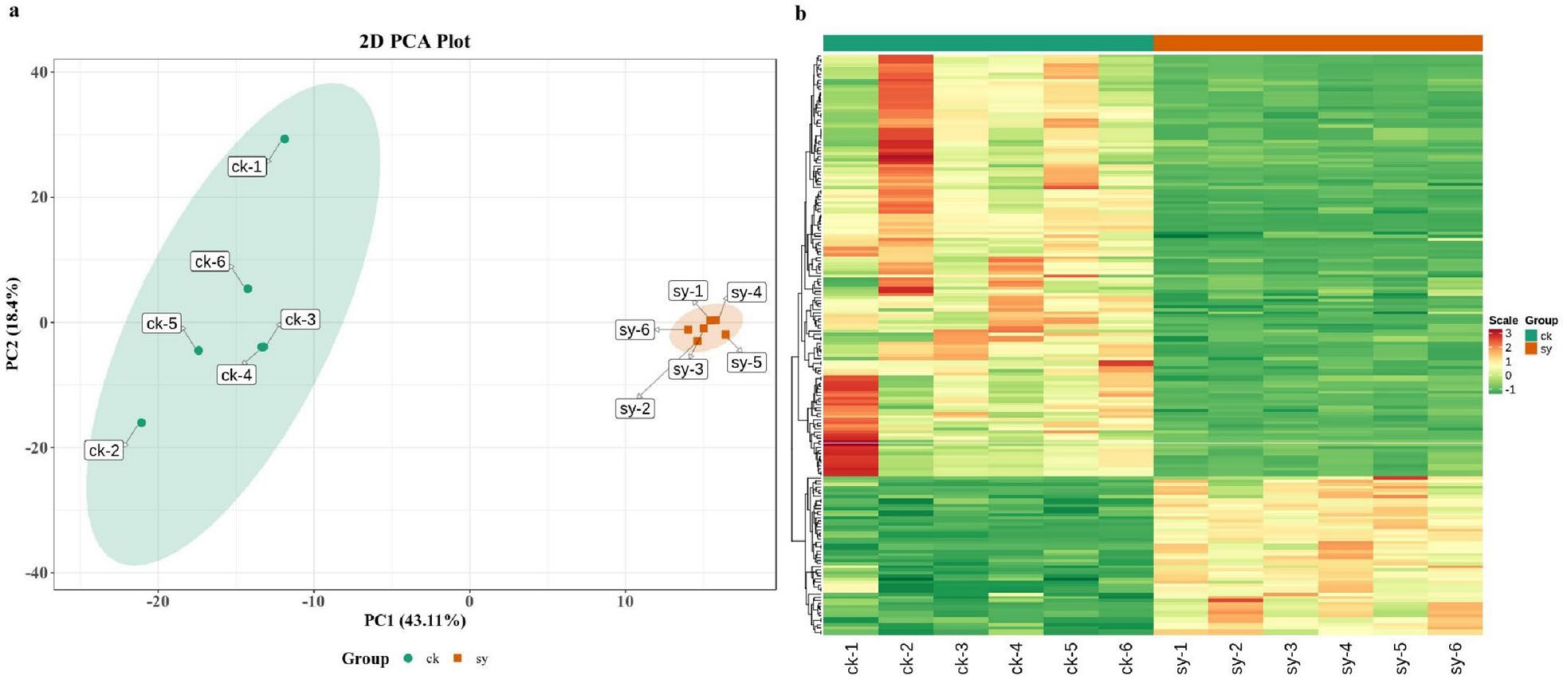
Metabolome profiling in *P. digitatum* in responding to X33 AMOP treatment. **a** Principal component analysis of metabolites accumulated in the control and X33 AMOP-treated samples. **b** Heatmap results of the metabolites accumulated in control and X33 AMOP-treated samples. Ck1 ~ ck6 represents the control group displayed six repetition. Sy1 ~ sy6 represents the X33 AMOP-treated group displayed six repetition

**Fig. 3:**
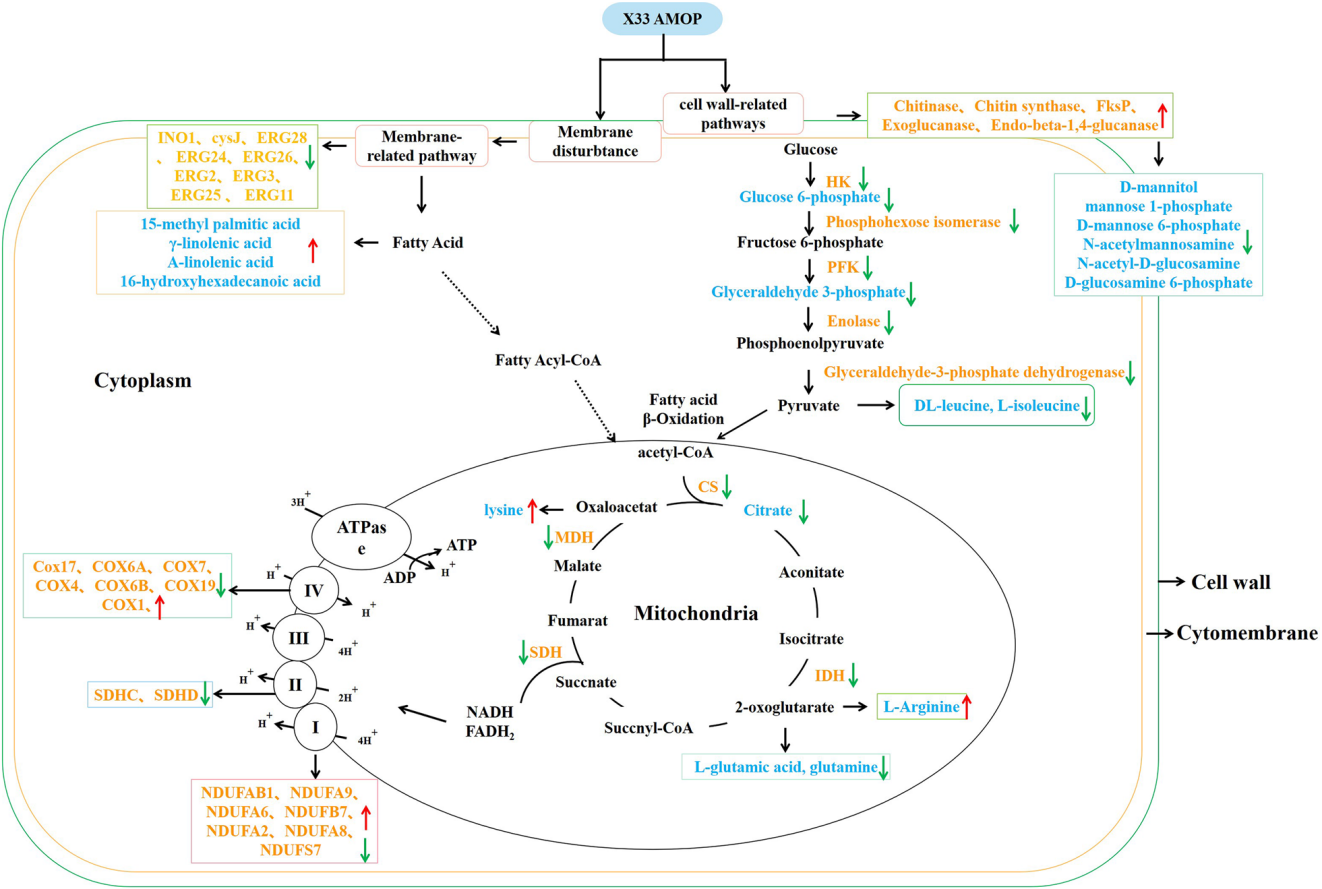
Accumulation of DEGs and differentially regulated metabolites in *P. digitatum* cells under 1.2 g/L X33 AMOP treatment. Metabolites marked with blue font indicate regulated in *P. digitatum* cells. Genes marked with yellow font indicate regulated in *P. digitatum* cells. Red arrows represent DEGs or metabolites were upregulated. Green arrows represent DEGs or metabolites were downregulated

To further explore the underlying restraining mechanism of X33 AMOP on *P. digitatum*, a metabolomic analysis was performed using LC–MS/MS. Principal component analysis (PCA) was used to evaluate the kinetic metabolome patterns of the two groups. Samples treated with X33 AMOP were distinct from the untreated samples, and the PCA results showed that PCA axes 1 and 2 accounted for 43.11% and 18.4% of the total variation, respectively. Therefore, X33 AMOP had a strong disturbance on the metabolomics of *P. digitatum* (Fig. 2a). To compare the discrepancies in the metabolite varieties and concentrations between the X33 AMOP-treated and control groups, the metabolites were clustered by Pearson correlation and the furthest neighbor method. The findings of these assays are in agreeance with those of the PCA (Fig. 2b). In total, 190 different metabolites were found in *P. digitatum* in the ck and sy groups. Among them, the concentration of 52 metabolites was upregulated and the biosynthesis of 138 metabolites was downregulated (Fig. 3). These metabolites mainly included phosphate sugars, amino acids, nucleotides, organic acids, and derivatives of the latter three (Table 3).

**Table 3:** Metabolites with statistical differences between X33 AMP-treated and untreated groups

Metabolites	Class	log2^FC^	Trend
Citric acid	TCA cycle	−1.09	Down
d-Glucose 6-phosphate	Glycolysis	−3.37	Down
Dl-Glyceraldehyde 3-phosphate	Glycolysis	2.38	Down
15-Methyl palmitic acid	Membrane structure	1.32	Up
γ-Linolenic acid	Membrane structure	1.09	Up
Α-Linolenic acid	Membrane structure	1.09	Up
16-Hydroxyhexadecanoic acid	Membrane structure	1.06	Up
d-Mannitol	Cell wall	−3.05	Down
Mannose 1-phosphate	Cell wall	−4.45	Down
d-Mannose 6-phosphate	Cell wall	−3.41	Down
n-Acetylmannosamine	Cell wall	−2.30	Down
n-Acetyl-d-glucosamine	Cell wall	−2.30	Down
d-Glucosamine 6-phosphate	Cell wall	−1.29	Down
DL-Leucine	Amino acid	−1.18	Down
l-Isoleucine	Amino acid	−1.23	Down
Lysine	Amino acid	1.94	Up
l-Glutamic acid	Amino acid	−2.03	Down
l-Cysteine	Amino acid	−1.18	Down
l-Arginine	Amino acid	1.01	Up
l-Histidine	Amino acid	1.28	Up
Glutamine	Amino acid	−1.64	Down
l-Glutamine	Amino acid	1.85	Up
l-Methionine	Amino acid	−1.96	Down
Cytosine	Genetic transmission	−2.01	Down
Hypoxanthine	Genetic transmission	−1.48	Down
Thymine	Genetic transmission	−1.55	Down
Guanine	Genetic transmission	−1.41	Down
d-Trehalose	Oxidative stress	−1.33	Down
d-sorbitol	Oxidative stress	−3.06	Down

### Validation of the expression of DEGs using qRT-PCR

**Fig. 4:**
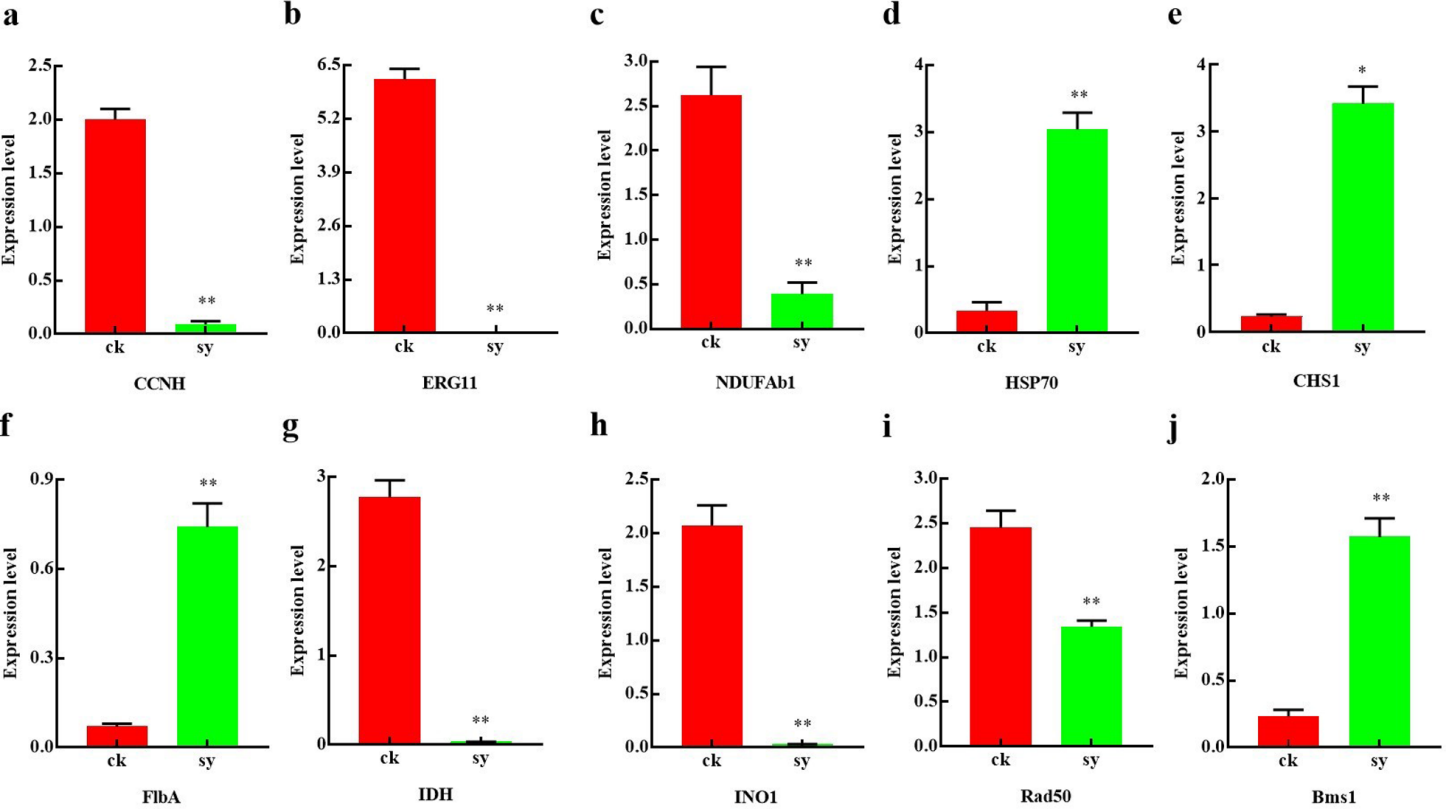
Relative expression levels profiling of candidate DEGs in responding to X33 AMOP treatment by qRT-PCR. The actin gene is used as the housekeeping gene. **a**. Relative expression levels of basal transcription factors CCNH; **b** Relative expression levels of 14-alpha sterol demethylase encoding ERG11 gene; **c** Relative expression levels of acyl carrier protein NDUFAb1; **d** Relative expression levels of heat shock protein 70; **e** Relative expression levels of chitin synthase A; **f** Relative expression levels of regulator FlbA; **g** Relative expression levels of isocitrate dehydrogenase; **h** Relative expression levels of myo-inositol-phosphate synthase; **i** Relative expression levels of DNA repair protein Rad50; **j** Relative expression levels of ribosome biogenesis protein

A total of 10 DEGs that were correlated to the major pathways were analyzed using qRT-PCR to validate the transcriptome results (Fig. 4). The transcriptional levels of genes, including *CCNH*, *Rad50*, *INO1*, *BMS1*, *FlbA*, *ERG11*, *NDUFAB1*, and *IDH* involved in the genetic transmission process, cell membrane, and energy metabolism in the treatment groups were repressed compared to those in the control group. Gene expression, including *HSP70* and *CHS1*, compared to stress response and cell wall, notably increased after X33 AMOP treatment. The expression patterns of these genes were consistent with the transcriptome profile (Table 4).

**Table 4:** RNA-seq analysis of ten genes mapped to the most enrichment pathways

Gene	Product	Description	Log2^(FC)^	Regulated type
XP_014534305.1	CCNH	Basal transcription factors	−1.00	Down
XP_014534778.1	Rad50	DNA repair protein Rad50	−1.29	Down
XP_014532480.1	INO1	Myo-inositol-phosphate synthase	−1.05	Down
XP_014532062.1	Bms1	Ribosome biogenesis protein (Bms1)	2.12	Up
XP_014538149.1	CHS1	Chitin synthase A	2.49	Up
XP_014538452.1	FlbA	Developmental regulator FlbA	3.85	Up
XP_014532172.1	ERG11	14-Alpha sterol demethylase Cyp51A	−2.62	Down
XP_014531425.1	NDUFAb1	Acyl carrier protein	−1.39	Down
XP_014536276.1	IDH	Isocitrate dehydrogenase	−1.47	Down
XP_014532714.1	Hsp70	Heat shock protein	1.62	Down

### Release of cell constituent after X33 AMOP incubation

**Fig. 5:**
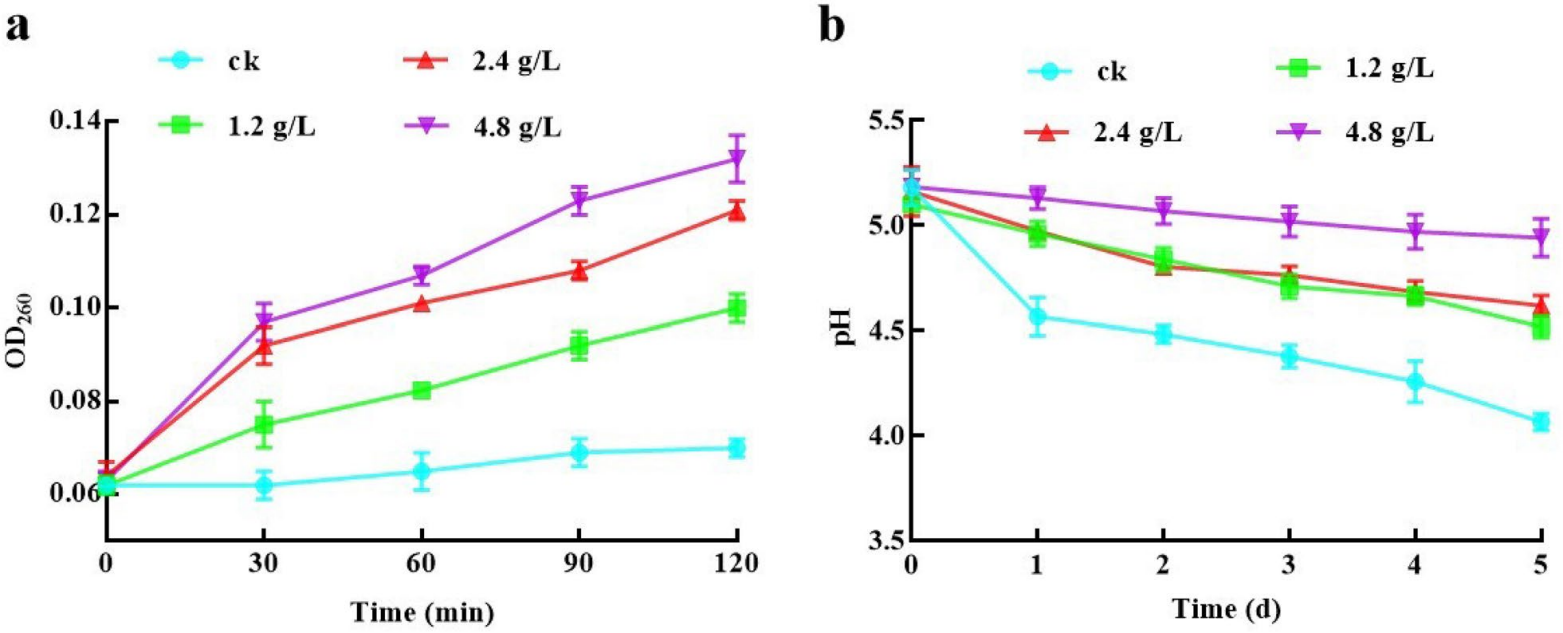
Effects of X33 AMOP treatment on growth status of *P. digitatum*. **a** X33 AMOP promotes the release of cellular constituents*.* **b** Inhibitory effect of the X33 AMOP on glucose-dependent acidification of medium in *P. digitatum*. Water cycle represents the control group treated by ultrapure water. Green square represents the treatment group incubated with 1.2 g/L of X33 AMOP. Red upper triangle represents the treatment group incubated with 2.4 g/L of X33 AMOP. Purple lower triangle represents the treatment group incubated with 4.8 g/L of X33 AMOP

Intracellular substances, including nucleic acids, are absorbed at 260 nm in suspensions and play a critical role in cell lifecycles (Huang et al. 2019). The optical density (OD) at 260 nm was used to assess the leakage of nucleic acids. As shown in Fig. 4a, the release of cellular elements was significantly elevated (*P* < 0.05) when *P. digitatum* was treated with X33 AMOP. The OD_260_ value in *P. digitatum* suspensions treated with 1.2 g/L of X33 AMOP for 0.5 h was 0.075, which was greater than that of the control group (0.062), and lower than the group incubated with 4.8 g/L of X33 AMOP (0.097). The OD_260_ value of the *P. digitatum* suspensions treated with 1.2 g/L of X33 AMOP maintained a steady ascending trend after 0.5 h of exposure, and that treated with 4.8 g/L continuously increased after 0.5 h of exposure and achieved a maximal absorbance of 0.132 after 2 h of exposure (Fig. 5a). The present study of constituents absorbing at 260 nm revealed that X33 AMOP disrupted the cytomembrane of *P. digitatum*, resulting in the release of cell constituents.

### Changes in external pH in *P. digitatum* after X33 AMOP incubation

Acidification of the external compartments involves ATP-dependent proton pumps inserted in the limiting cytomembrane. Various metabolic functions might also be influenced by acidification, ultimately inducing apoptosis. In the present study, we analyzed the probability that the cytomembrane limited this acidic external resorbing compartment. As shown in Fig. 4b, the test groups treated with 4.8 g/L X33 AMOP decreased the pH value from 5.18 to 4.94 after 1 h of incubation, whereas the pH value of the control group decreased from 5.18 to 4.07. High X33 AMOP concentrations effectually inhibited such acidification compared to the control. The inhibitory activity was increased with elevating X33 AMOP concentration and incubation time; therefore, the energy synthesis and metabolism of *P. digitatum* might be affected by X33 AMOP treatment (Fig. 5b).

### Effect of X33 AMOP treatment on changes in the energy metabolism of *P. digitatum*

**Fig. 6:**
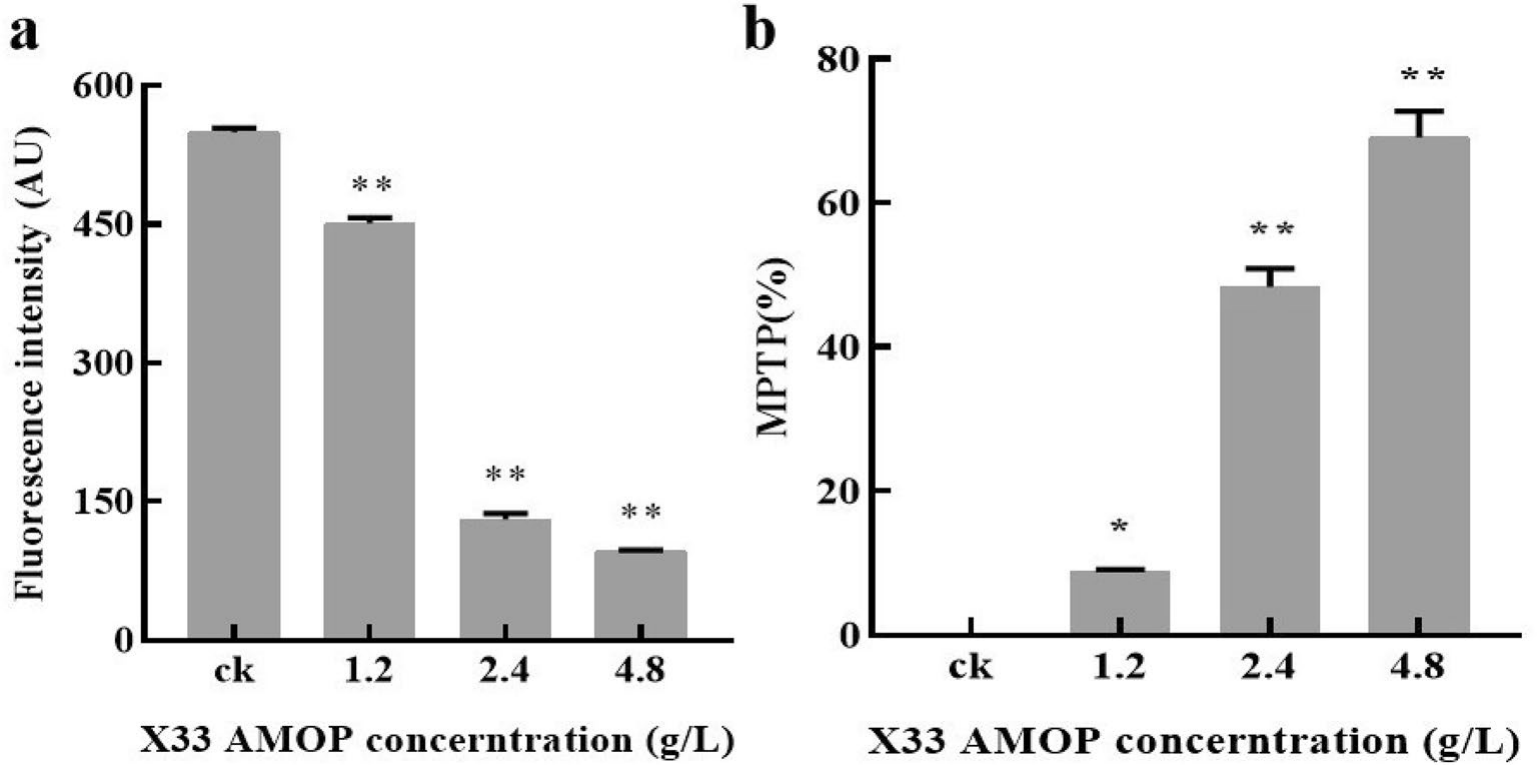
Effects of X33 AMOP treatment on mitochondrion damage of *P. digitatum*. **a** Effect of X33 AMOP on MMP of *P. digitatum*. **b** Effect of X33 AMOP on MPTP of *P. digitatum*. The data were shown as the mean ± SD (*n* = 3), ∗ *p* < 0.05, ∗  ∗ *p* < 0.01

Reactive oxygen species (ROS) can cause mitochondrial membrane permeabilization after energy metabolism disturbances. Thus, we evaluated the depolarization of the MMP using a fluorescent dye (RH123). After treatment with gradient X33 AMOP concentrations, the intensity of RH123 fluorescence decreased in a dose-dependent manner (Fig. 6a). The fluorescence intensity of MMP treated with 4.8 g/L X33 AMOP was dramatically reduced to 97.17 au (arbitrary units), which was 17.67% of that of the control (549.78 au). Exposure to X33 AMOP also led to a marked change in the MPTP opening (Fig. 6b). The MPTP opening varied up to 69.18 ± 5.86% after the hyphae were treated with 4.8 g/L X33 AMOP for 1 day, which was greater than that of the control at 4.42 ± 0.04%. The MPTP opening was increased after X33 AMOP treatment, and the opening of MPTP was aggravated with increasing X33 AMOP concentration. Therefore, the mitochondria might participate in the apoptosis of *P. digitatum* cells induced by X33 AMOP.

### Effect of X33 AMOP treatment on the biosynthesis of nucleic acids in *P. digitatum*

**Fig. 7:**
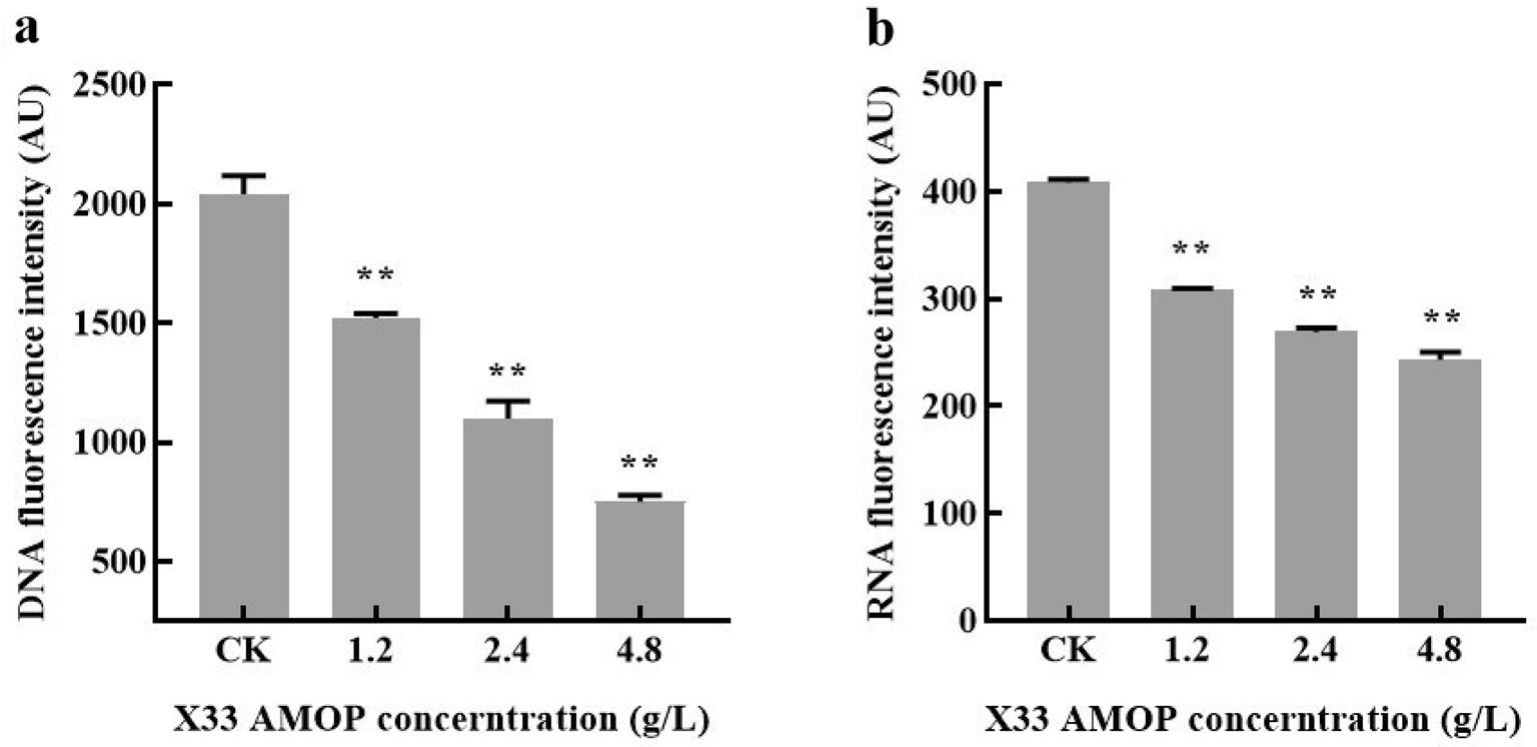
Effects of X33 AMOP treatment on the nucleic acid degradation of *P. digitatum*. **a** X33 AMOP induced DNA degradation. **b** X33 AMOP induced RNA degradation. DAPI was used as a fluorochrome for the indirect measurement of DNA and RNA concentration. The data were shown as the mean ± SD (*n* = 3), ∗ *p* < 0.05, ∗  ∗ *p* < 0.01

DAPI is a nucleic acid-binding fluorescent dye that can be embedded in nucleic acids. After binding with nucleic acids, different fluorescence intensities can be detected, corresponding to different nucleic acid concentrations. To study the potential influence of X33 AMOP on the biosynthesis of nucleic acids in *P. digitatum*, a DAPI dye was applied to quantify the DNA and RNA content. The results indicated that the DNA fluorescence intensity was 751.86 when incubated with 4.8 g/L of X33 AMOP, which was 63.14% lower than that of the control (2039.63) (Fig. 7a). The RNA fluorescence intensity was 243.69 when incubated with 4.8 g/L of X33 AMOP, which was 40.36% lower than that of the control (408.57) (Fig. 7b). This decrease in fluorescence intensity indicated that the biosynthesis of nucleic acids was a potential target for X33 AMOP against *P. digitatum*.

## Discussion

The present study aimed to research the potential antifungal mechanism of X33 AMOP against *P. digitatum* using an omics analysis strategy. Integration analysis of transcriptomics and metabolomics was performed to acquire novel insights into the antimicrobial mechanism of X33 AMOP and indicate the molecular mechanism underlying *P. digitatum* death.

### X33 AMOP incubation induced cytomembrane virulence in *P. digitatum*

The cytomembrane plays an essential role in maintaining cell viability owing to the defensive function of separating the cell from its surroundings (Li et al. 2020). Transcriptome analysis revealed that the expression of *glnA*, *FAS1*, *FAS2*, *ERG6*, and *ERG27* was increased by approximately 1.08- to 1.85-fold, whereas the expression of *INO1*, *ERG2*, *ERG3*, *ERG11*, *ERG24*, *ERG25*, *ERG26*, *ERG28*, and *cysJ* was downregulated by approximately 1.41- to 2.62-fold in *P. digitatum* after X33 AMOP incubation. These genes were associated with cell membrane-related pathways, such as fatty acid and steroid synthesis, inositol phosphate metabolism, and unsaturated fatty acid biosynthesis, indicating that irreversible damage occurred in the cell membrane structure of *P. digitatum*. A similar result has been reported in the recent study that flavonoids inhibited phytopathogen blue mold (*P. italicum*) by interrupting cell membrane structures (Luo et al. 2020). Our previous study also showed a similar cell wall damage action mechanism (Lin et al. 2020). In the present study, an enhancement in cytomembrane-related metabolites, including 15-methyl palmitic acid, γ-linolenic acid, α-linolenic acid, and 16-hydroxyhexadecanoic acid, was observed to change the fatty acid ratio, and thus change the permeability of the cytomembrane. This is consistent with a previous study that showed that cell apoptosis in the fungus was primarily caused by the hydrolysis of glycerophospholipids to elevate the permeability of cell membranes under heavy oxidative stress (Chen et al. 2019). The addition of X33 AMOP caused a rapid loss of the absorbing substance at 260 nm in the assayed fungal suspensions. The maximum release of cellular elements was found in the *P. digitatum* cell suspensions treated with 4.8 g/L of X33 AMOP, in agreeance with the transcriptome analysis data. This result showed downregulation of the expression of genes correlated to cell membrane compositions, consistent with a previous study that reported that C_12_O_3_TR retarded the growth of *P. digitatum* by changing the expression of genes encoding ergosterol metabolism and fatty acid biosynthesis (Li et al. 2020).

### X33 AMOP incubation induced cell wall damage in *P. digitatum*

Glucan, chitin, chitosan, and glycoproteins form the key structure of fungal cytoderms, and play a pivotal role in protecting the cell and controlling cellular permeability (Guo et al. 2020). Results from the present study found that several pathways, including amino sugar, nucleotide sugar, starch, and sucrose metabolism, involved in cell wall biosynthesis were influenced by X33 AMOP incubation. *CHIA1*, *CHIB1*, *CHS1*, *FKSP*, and *CBH1* expression was upregulated; therefore, the integrity of the cell wall might have been disrupted. The upregulated expression of genes concerning the cell wall might be caused by the defensive response or the compensatory mechanism of cells to overcome the external stress. A previous study showed a similar result, reporting that limonene treatment induced the upregulation of several genes related to the cell wall integrity signaling pathway (Brennan et al. 2013). Metabolites involved in the biosynthesis of cell walls, such as d-mannitol, mannose-1-phosphate, d-mannose-6-phosphate, N-acetylmannosamine, N-acetyl-d-glucosamine, and d-glucosamine-6-phosphate that influence the biosynthesis of glucan and chitin, are detrimental to the biosynthesis of *P. digitatum* cell walls. This result is consistent with the effect of 2-methoxy-1,4-naphthoquinone on the postharvest fungal pathogen, *P. digitatum* (Guo et al. 2020).

### X33 AMOP incubation induced abnormal energy metabolism in *P. digitatum*

Fungal cells decrease their external pH after glucose addition because of the proton efflux of the plasma membrane proton-pumping ATPase Pma1 (Ma et al. 2015). In the acidification assay, X33 AMOP significantly suppressed the glucose-induced decrease in external pH at 4.8 g/L. Such effect can also be induced by some fungicides at the same concentrations (Lin et al. 2020), suggesting that the proton-pumping ability of X33 AMOP might support its antifungal action mode. The citrate cycle is a central pathway for oxidative phosphorylation in cells and fulfills their bioenergetic, biosynthetic, and redox balance requirements. Most genes encoding the corresponding enzymes, such as citrate synthase, isocitrate dehydrogenase, and malate dehydrogenase (NAD-dependent), listed in the citrate cycle pathway were downregulated and caused an obstacle in citrate biosynthesis. This result was consistent with that of the above study, suggesting that X33 AMOP incubation could disrupt mitochondrial function and inhibit the citrate cycle of *P. digitatum*. A previous study also showed that limonene could damage the mitochondrial membrane permeability and destroy the citrate cycle pathway and oxidative phosphorylation of *P. digitatum* (Zhang et al. 2020). Marked changes in genes related to oxidative phosphorylation were also observed in the present study. For instance, the following genes were repressed: *NDUFA2*, *NDUFA6*, *NDUFA8*, *NDUFA9*, *NDUFAB1*, *NDUFB7*, and *NDUFB9*, all of which constitute the mitochondrial complex I; SDHC and SDHD, which belong to mitochondrial complex II; *COX4*, *COX6A*, *COX6B*, *COX7*, *COX17*, and *COX19*, which belong to mitochondrial complex IV; and the ATP synthase delta chain, which is part of the mitochondrial complex V (OuYang et al. 2018). Conversely, *COX1* and *Ndufs7* expression was increased by 1.89- and 2.86-fold. A previous study reported a similar phenomenon where tea tree oil treatment produced several proteins related to glycolysis and the citrate cycle pathway, thereby inducing mitochondrial dysfunction and disrupting energy metabolism (Xu et al. 2017).

On the mitochondrial inner membrane, a proton pump can extract protons in the matrix into the membrane gap and accumulate many protons, causing the mitochondrial membrane gap to accumulate many positive charges (Ma et al. 2020). Nevertheless, many negative charges were produced in the matrix, leading to marked changes on both sides of the mitochondrial inner membrane and, thus, the generation of transmembrane potential (Zorova et al. 2018). Normal MMP is a prerequisite for the maintenance of oxidative phosphorylation and ATP generation in the mitochondria. Loss of this mitochondrial function and cell death is linked to MMP loss due to an increase in membrane permeability, which is frequently termed the mitochondrial permeability transition (Vadim et al. 2021). Depending on the severity of stress, permeability transition can be presented as a large MPTP, which is a cell-lethal, high-conductance MPTP, or as low-conductance mitochondrial ion/proton leak (Vadim et al. 2021). In the present study, we found that X33 AMOP aggravated the MPTP opening and caused MMP loss in *P. digitatum*, suggesting the existence of irreversible mitochondrial membrane damage. Mitochondrial injury promotes ROS generation, and excessive ROS causes rapid oxidation of various biomolecules, including nucleic acid, proteins, and lipids, in the nucleus and mitochondria, causing cell death. Hence, the disturbance of mitochondrial function indirectly supported the downregulation of DEGs associated with the energy metabolism process. Tea tree oil has also been shown to induce mitochondrial dysfunction, decrease ATP content, and suppress the citrate cycle of *B. cinerea* (Xu et al. 2017)*.*

The downregulation of most genes involved in energy-related routes can be attributable to two factors. X33 AMOP might cause cellular dysfunction, resulting in a suppressive effect on respiration and energy metabolism. In addition, the reduction in energy metabolism may be feedback or a response to adverse environmental factors to maintain cell activity. In the present study, several genes, such as *hsp 42* and *hsp 70*, which are associated with the heat shock protein, a crucial molecular chaperone of the stress reaction, were overexpressed by 1.41- to 1.62-fold. A previous study showed that *P. digitatum* genes responding to stress, including *hsp70*, *hsp90*, *hsp78*, and *hsp60*, were overexpressed after thanatin treatment (Feng et al. 2020). Furthermore, D-sorbitol levels in *P. digitatum* cells decreased after X33 AMOP treatment. Sorbitol, a compatible solute, is a common polyhydric alcohol that may also protect intracellular enzyme activity and cytomembrane integrity via its ROS scavenging activities (Han and Yuan 2009). These results support our proposed ROS burst in *P. digitatum* mycelium, which is consist with our previous study (Lin et al. 2020).

### X33 AMOP incubation induced disturbance of the genetic transmission process in *P. digitatum*

To alleviate the unfavorable growth conditions caused by X33 AMOP treatment, *P. digitatum* develops relative responses by adjusting its gene expression program. In the present study, considerable alterations in gene expression levels correlated with genetic transmission were found in *P. digitatum*. Genes related to nucleotide excision repair, DNA replication, aminoacyl-tRNA biosynthesis, mRNA surveillance, basal transcription factors, RNA degradation, and RNA transport pathways were partly inhibited, suggesting a lesion in the translational activity of cells. For instance, eight DEGs were related to the mRNA surveillance pathway, among which seven were downregulated by 1.54- to 2.48-fold. A consistent case was found in *P. expansum* cells treated with cinnamaldehyde and citral combination (Wang et al. 2018c). Moreover, purines and pyrimidines were related to nucleic acid synthesis, energy supply, and stress tolerance. Cytosine, hypoxanthine, thymine, and guanine levels were downregulated by 1.34- to 2.65-fold, suggesting an abnormality in nucleotide-related metabolism. Purine metabolism is an essential part of cell physiology and is involved in various aspects of cell metabolism, whereas hypoxanthine is a pivotal component of the cellular nucleotide pool in purine metabolism (Shu et al. 2020).

Based on the above data, the potential action of X33 AMOP in the genetic transmission process was verified. Previous research has shown that spore germination and hyphae growth cultivated at favorable nutritional environments are commonly synchronized with multiple metabolic activities, such as respiration, nucleic acid, and protein biosynthesis (Zhou et al. 2018). In the present study, the DNA and RNA content of *P. digitatum* was reduced by X33 AMOP incubation (Fig. 6a); therefore, X33 AMOP suppressed DNA and RNA biosynthesis in *P. digitatum*. The above results are consistent with our previous study, that is, X33 AMOP interfered with the normal translation of *P. digitatum* mycelium, and X33 AMOP disrupted the transcription process of *P. digitatum.*

### X33 AMOP incubation induced disturbance of amino acid metabolism in *P. digitatum*

Apart from the cell wall, cytomembrane, and mitochondrial respiratory chain, and genetic transmission changes, *P. digitatum* also underwent amino acid metabolism change in the presence of X33 AMOP. Microorganisms frequently activate certain amino acid biosynthetic pathways to survive when undergoing multiple stress responses. In the present study, the content of several amino acids such as DL-leucine, l-isoleucine, l-glutamic acid, l-cysteine, was changed in the X33 AMOP treatment group compared to the control. Although amino acids are decomposed and regenerated by disparate approaches, they are all converted into five metabolites that flow into the citrate cycle and eventually oxidize to carbon dioxide and water. The carbon skeletons of cysteine, leucine, and lysine are converted into acetyl-CoA; those of arginine, histidine, glutamate, glutamine, and l-glutamine are converted to α-ketoglutaric acid; and those of isoleucine and methionine are converted into succinyl coenzyme A (Ma et al. 2020). The results from the present study indicated the adjustment of osmotic balance and protecting cells from outside pressure was damaged in *P. digitatum* after X33 AMOP treatment.

## Conclusion

In summary, this study reveals the antifungal mechanism of X33 AMOP against *P. digitatum* by using transcriptomic and metabolomic techniques. X33 AMOP is capable of induce mitochondrial dysfunction, decrease the efficiency of energy metabolism in mitochondria, induce ROS overproduction, disrupt cell integrity and genetic information delivery, finally suppress the growth of *P. digitatum*. The present study provides a better understanding of the transcriptome and metabolic differences between untreated and treated with X33 AMOP and a theoretical basis for the antifungal activity of X33 AMOP against this pathogen.

### Supplementary Information

**Additional file 1.** The primers used in this study and the structure identification of X33 AMOP.

## Data Availability

All data are included in this article.

## Abbreviations

AMPs: Antimicrobial peptides
DAPI: 4,6-Diamidino-2-phenylindole
DEGs: Differentially expressed genes
MMP: Mitochondrial membrane potential
MPTP: Mitochondrial permeability transition pore
ROS: Reactive oxygen species
VIP: Variable importance in the projection
X33 AMOP: X33 antimicrobial oligopeptide
