# Supplementary material for: Combined transcriptome and metabolome analyses reveal the potential mechanism for the inhibition of Penicillium digitatum by X33 antimicrobial oligopeptide
Source: Bioresour Bioprocess. 2021 Dec 2;8(1):120. doi: 10.1186/s40643-021-00472-5 (PMC10991954; doi:10.1186/s40643-021-00472-5)
Supplement: Supplementary file 1 — Additional file 1. The primers used in this study and the structure identification of X33 AMOP. [file 40643_2021_472_MOESM1_ESM.doc]

Additional file 1

Table s1. Primer sequences for gene amplification by qRT-PCR

| Gene | Forward primer | Reverse primer | Product |
| --- | --- | --- | --- |
| XP_014534305.1 | GGGGTTCATTTCCCGGGCC | AGGCAGATTGCACCCGTT | CCNH |
| XP_014534778.1 | CGGTCCGACAACGAGAATGC | GCCAGAACCTTTTGGCCTG | Rad50 |
| XP_014532480.1 | TTGGATGCTGCCAATGGCGC | CAGCTTGTTGTCCGCGGTGC | INO1 |
| XP_014532062.1 | CGCTCGCGTGGCTATACCCG | GTCGAGCTCCCCTTCGTCAT | Bms1 |
| XP_014538149.1 | ACCCGTTCGGAGACGAGC | AGATGGCATGGGAAGATGCGA | CHS1 |
| XP_014538452.1 | CGGAGGATGAGCGACCGT | AGGCATGCGGTTGGACTG | FlbA |
| XP_014532172.1 | CCGTTCGGGGCCGGTAG | AGACGAGTAATCCGTGTCTGGC | ERG11 |
| XP_014531425.1 | CCGCTCCGCCATTGTTCG | TCAGACCAGCAGGAGCGG | NDUFAb1 |
| XP_014536276.1 | AACACCGATGAGTCCATCGCTGG | GAGACGGTGCTCGTACCAGATG | IDH |
| XP_014532714.1 | GCCAAGAACCAGGCCGCC | CAACGGGGTTGCCACCCT | Hsp70 |

The purification process of X33 AMOP:

The fermentation broth was collected and eluted with D101 macroporous adsorption resin chromatography and C18 column chromatography. The elution system was eluted with 0.01 mol/L HCl aqueous solution and pure water at a flow rate of 1 drop/s. TLC detection and activity tracking were carried out to collect the extract of the active substances. The crude metabolite was freeze-dried to obtain powdered active substance, which was weighed to determine its quality and stored at 4^o^C. The X33 antifungal metabolite from *S. lavendulae* strain X33 was diluted with sterile water into different concentrations for further experiments. we have preliminarily deduced the structure of compound 1 (Fig. S1).

The derivation processed are as follows and the relevant raw data is at the end of this article: Compound 1, the carbon spectrum shows that there are 13 carbon signals, 8 methylene in DEPT-135 spectrum, and 2 methenyl groups DEPT-90 spectra (Fig.S2, Fig. S3, Fig S4). Compared with the carbon spectra, carbon atoms at δ171.8 and 171.6 are sp2 hybridization and belong to carbonyl group. The infrared spectrum also shows it is a carbonyl carbon. According to intensity ratio of the methylene which is 2:1 at chemical shift 24.2 and 22.9, 29.4 and 29.1, 36.6 and 36.5, 38.7 and 38.9 , those of methine at 48.6 and 48.9 is 2:1, and signal strength of quaternary carbon at 171.8 is greater than those at 172.1. We speculated that compound may be polymerized composed of some same monomer. It was hydrolysed in 6mol/L HCl . The Rf value and the rotation direction of the optical rotation of the hydrolysis product were the same as those of the standard lysine , indicating that the compound was polylysine. According to the integral area of H nuclear magnetic resonance spectrum, it shown the compound is composed of 3 lysine residues. The negative ion mold HR-EMS spectrum gave the molecular weight of 402.2911[M]- , calculated value 402.2954 and 401.2879[M-H]- calculated value 401.2876. The formula C18H38N6O4 (Fig. H) was confirmed by MS-MS spectra. The HSQC spectra show that the peak of the hydrogen at δ3.53 is related to that of the carbon at δ 48.7, indicating that they form a methylene (Fig. S6). There was a cross point at the peak of the hydrogen at δ3.53 and that of the carbon at δ 171.8 in HMBC spectrum (Fig. S7). Through the biuret test it cannot react. This shows that ϵ - amino of lysine and carbonyl condense ϵ - three polylysine. 1H NMR spectral data (D2O, 400 M) : δ 1.51 (6H, m), 1.62 (4 H, m), 1.66 (4 H, m), 2.60-2.54, 2.93 (2H, t, J = 4 Hz), 3.12 (6H, t, J = 8 Hz), 3.22 (4H, s), 3.53 (3 H, m)(Fig. S5), 13C NMR spectral data (D2O, 100 M) : δ 172.1 (s), 171.8 (s), 49.2 (d) and 48.9 (d) and 38.9 (t), 38.7 (t), 36.8 (t), 36.7 (t), 29.5 (t), 29.1 (t), 24.3 (t) (t) 22.9. The fragmentation law of mass spectrometry is also consistent with the fragment ions of tripolysine (Fig. S9). In addition, the energy spectrum data also show that the substance contains the element C, O, N (Fig. S10.). To sum up, the substance is  ϵ - three polylysine. (C_18_H_38_N_6_O_4_).

Fig. S1. Structural Formula of compound 1

In addition, please allow me to explain that the identification of the main component is the unpublished primary result. By the way, please allow us to explain that this work belongs to another student, we can't include it as supplementary material until he publishes it. While, in the following time, he will publish relevant content successively if get the deep results. Thanks for the reviewer’s reminding. We have added the reference into the manuscript/ please check it.

The chemical structure(s) of the antifungal metabolite is shown in 2 part of Reviewer #1 comments and its data is the fellow:


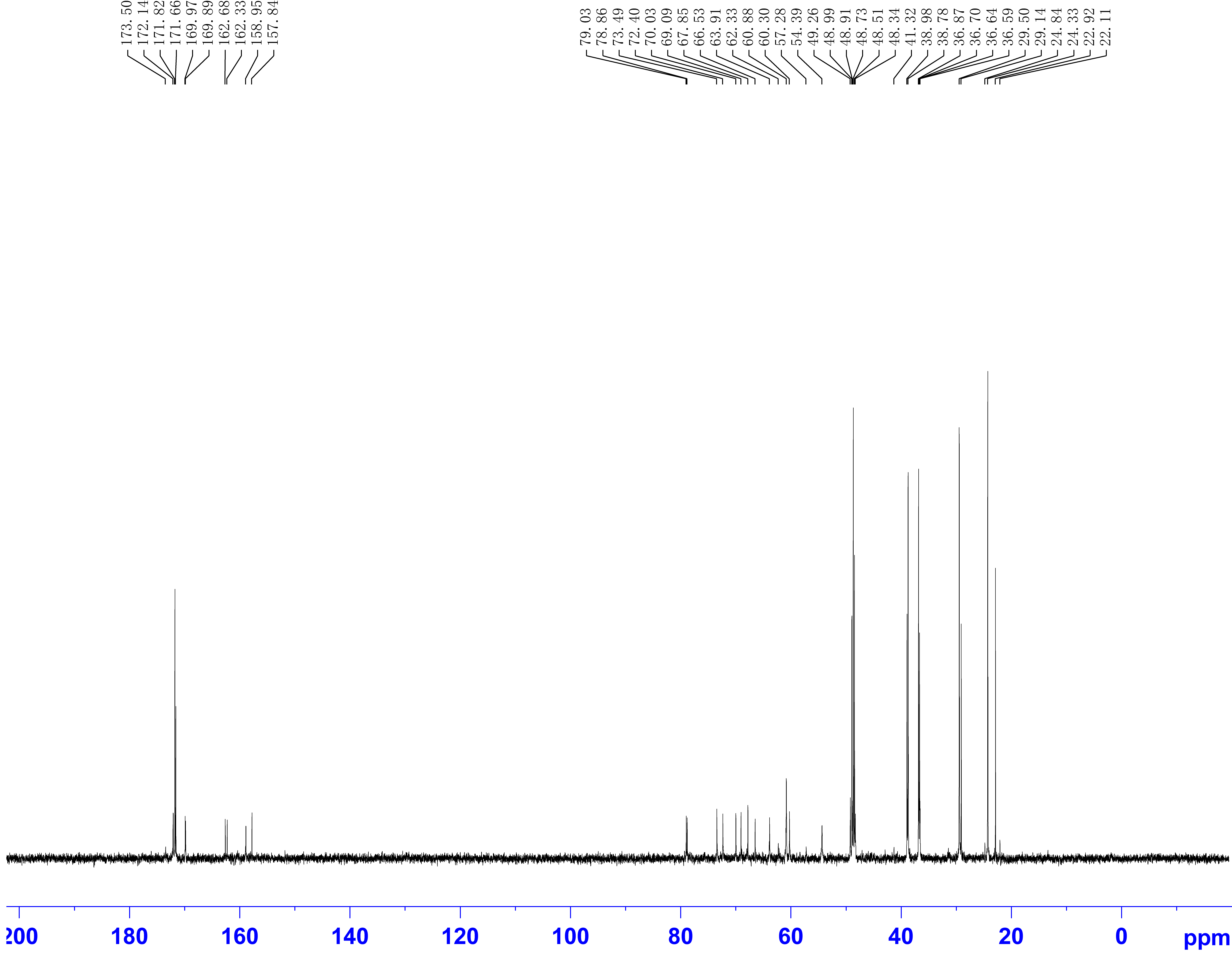


Fig. S2. Carbon nuclear magnetic resonance of compound 1


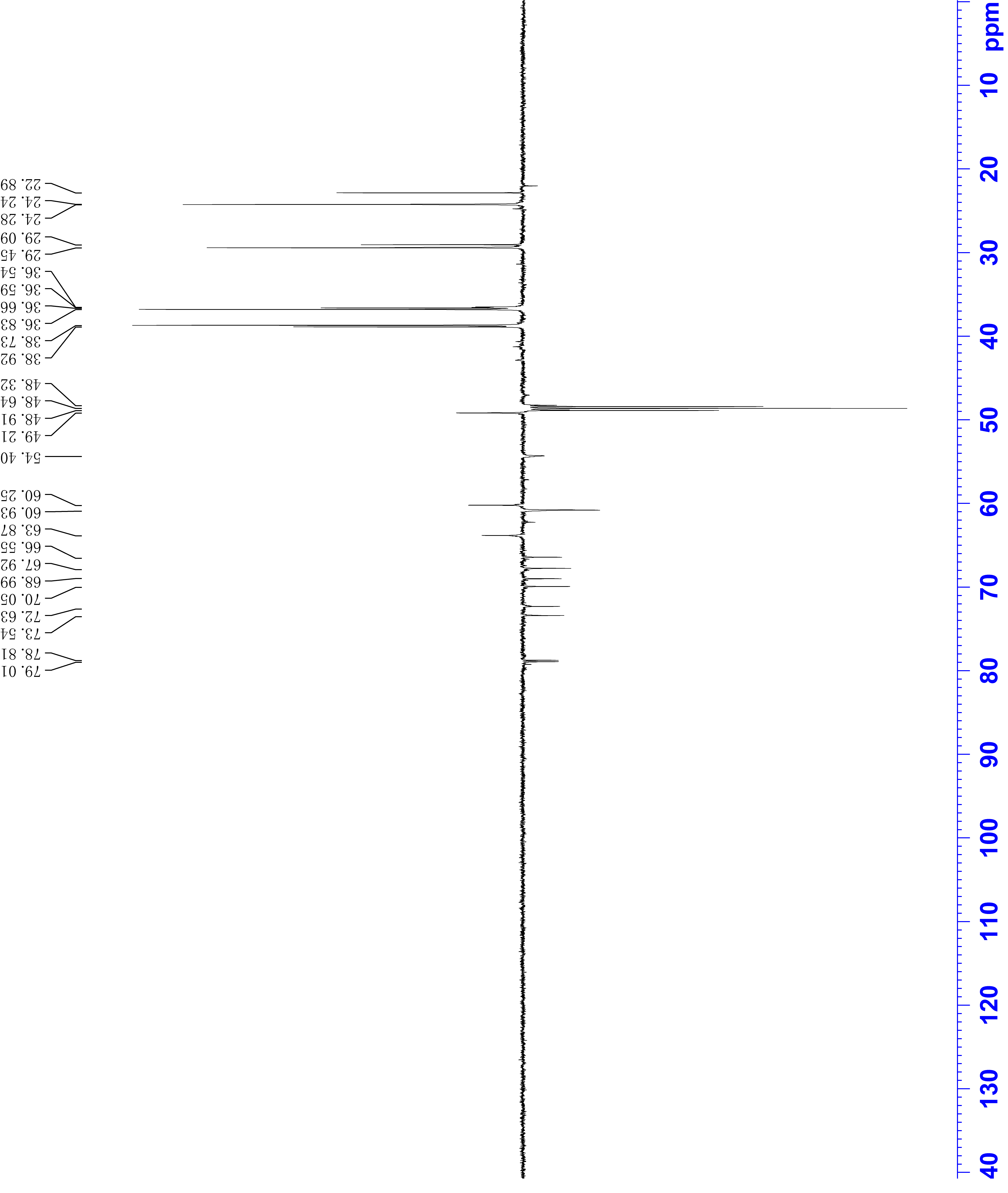


Fig. S3. DEPT-135 spectrum of compound 1


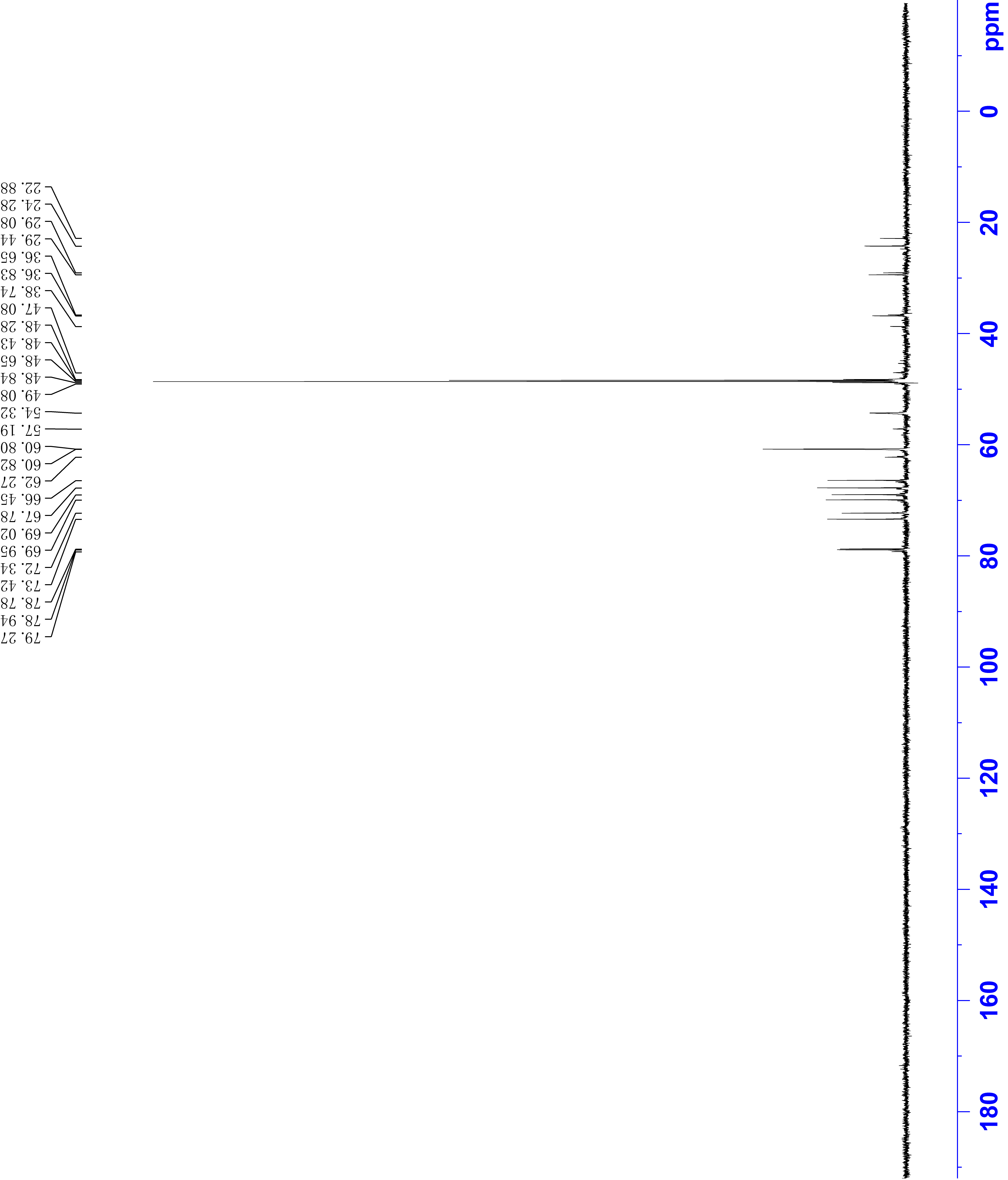


Fig. S4. DEPT- 90 spectrum of compound 1


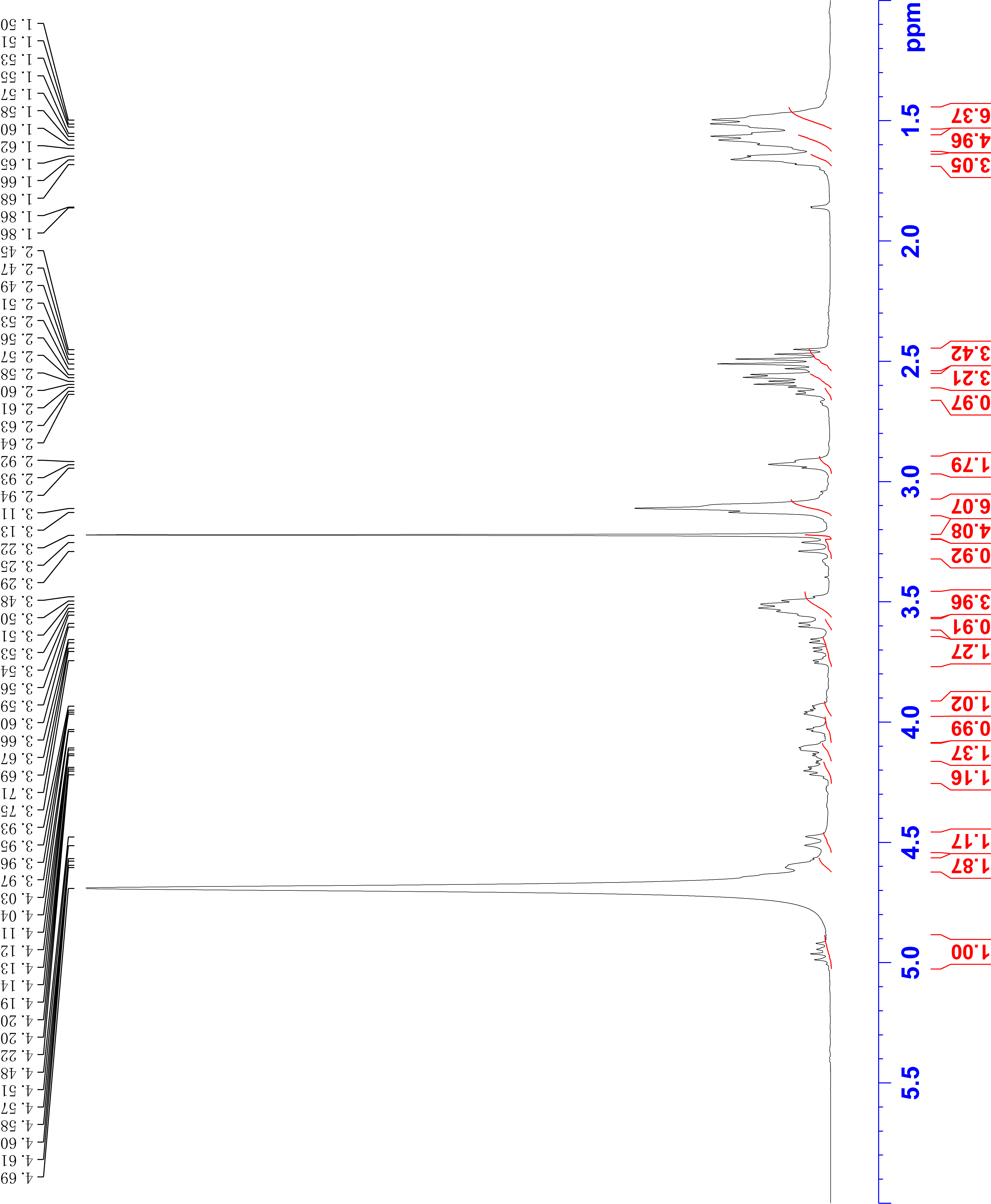


Fig. S5. H nuclear magnetic resonance spectrum of compound 1


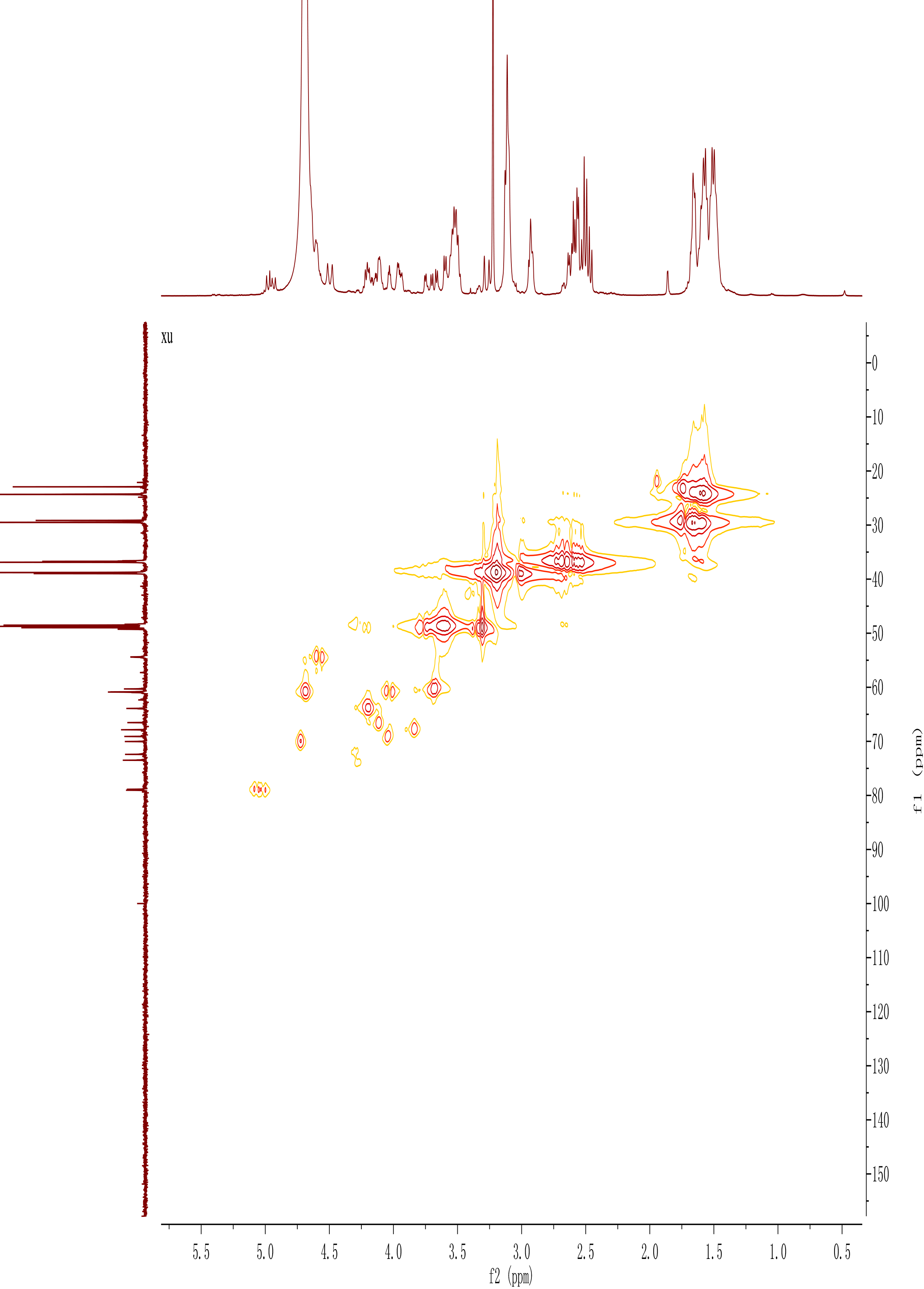


Fig. S6. HSQC spectra of compound 1


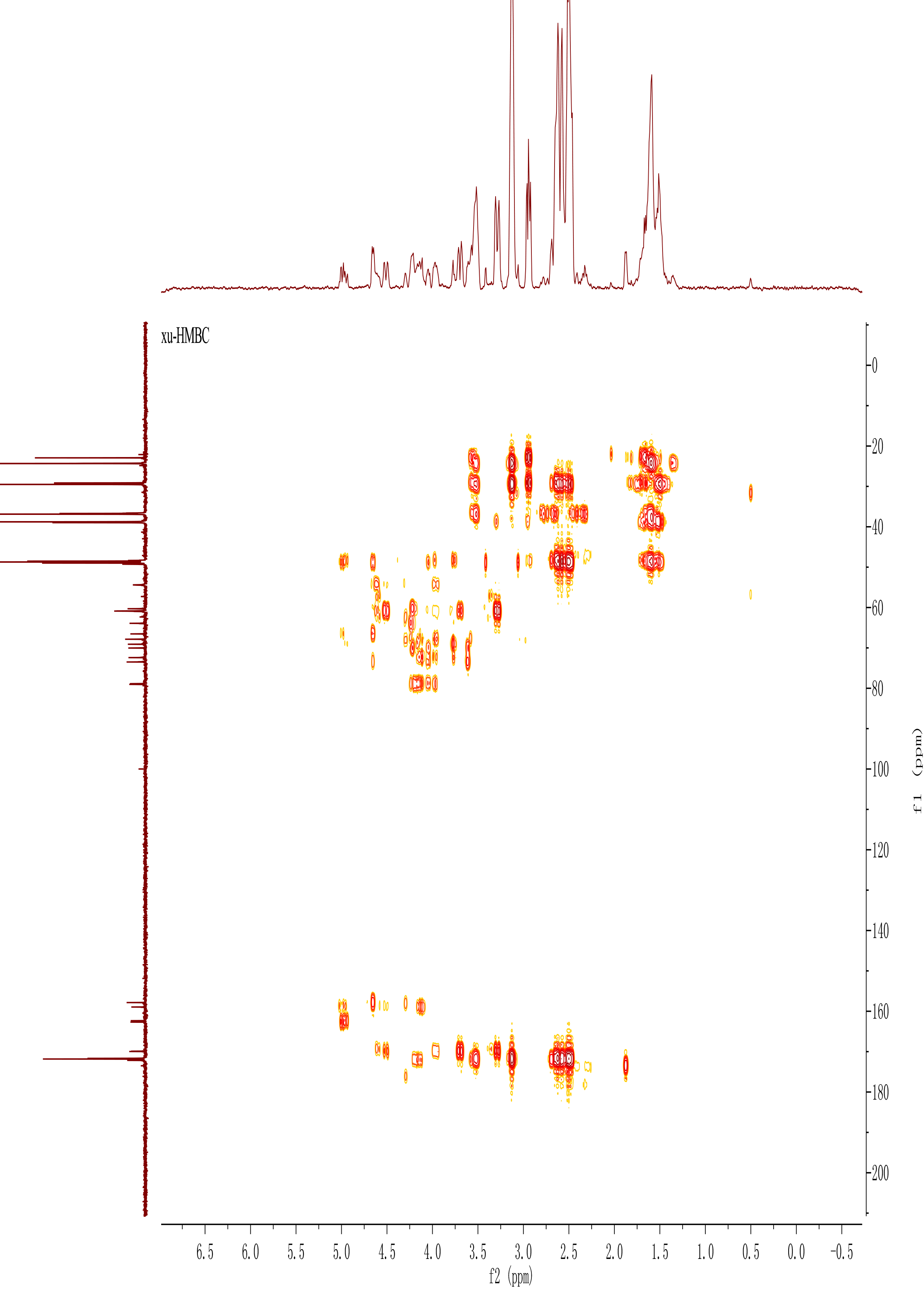


Fig. S7. HMBC spectrum of compound 1


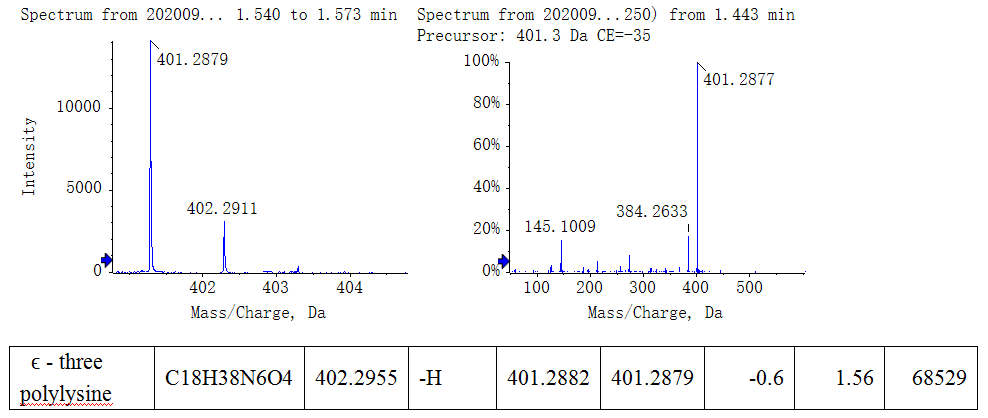


Fig. S8. mass spectrometry of compound 1

Fig. S9. mass spectrometry of compound 1


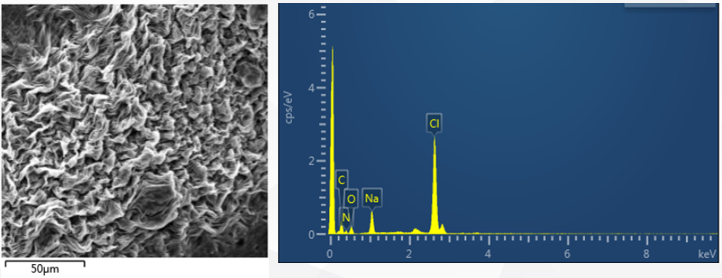


| element | Line type | Apparent concentration | K ratio | wt% | wt% Sigma | Standard sample label |
| --- | --- | --- | --- | --- | --- | --- |
| C | K-line | 1.88 | 0.01880 | 46.39 | 2.35 | C Vit |
| N | K-line | 2.34 | 0.00417 | 16.48 | 2.77 | BN |
| O | K-line | 1.71 | 0.00577 | 14.53 | 1.16 | SiO2 |
| Na | K-line | 2.29 | 0.00965 | 5.29 | 0.31 | Albite |
| Cl | K-line | 7.95 | 0.06945 | 17.31 | 0.84 | NaCl |
| Total: |  |  |  | 100.00 |  |  |

Fig. S10. Energy spectrum of compound 1
